# Phylogenetic and molecular insights into the genetic diversity of the invasive nematode *Ashworthius sidemi* infecting Poland's free-ranging European bison (*Bison bonasus*)

**DOI:** 10.1016/j.ijppaw.2026.101259

**Published:** 2026-07-04

**Authors:** Magdalena Świsłocka-Cutter, Anetta Borkowska, Rafał Kowalczyk, Marta Kołodziej-Sobocińska

**Affiliations:** a Department of Zoology and Genetics, Faculty of Biology, University of Białystok, Ciołkowskiego 1J, Białystok, 15-245, Poland; b Mammal Research Institute, Polish Academy of Sciences, Stoczek 1, Białowieża, 17-230, Poland

**Keywords:** Ashworthius sidemi, European bison, Invasive parasite, Molecular markers, Trichostrongylidae

## Abstract

The invasive blood-sucking nematode *Ashworthius sidemi*, originally parasitising Asian cervids, has spread across Europe following human-mediated introductions of sika deer (*Cervus nippon*). In this study, we characterised the molecular variation of *A. sidemi* obtained from two neighbouring populations of European bison (*Bison bonasus*) in northeastern Poland, located in the Białowieża Primeval Forest and Knyszyńska Forest. Individual worms (one per host) were analysed using nuclear molecular markers (18S rRNA, 28S rRNA, ITS-1) and mitochondrial markers (COX1, ND4). All analysed markers revealed no detectable intraspecific genetic variability among the 16 specimens sequenced, with a single haplotype identified for each marker. This finding is consistent with a founder effect and/or recent introduction of the parasite. The genetic homogeneity observed across geographically close bison herds may also reflect host mobility. Phylogenetic analyses based on both nuclear and mitochondrial markers consistently placed *A. sidemi* within the subfamily Haemonchinae, closely related to *Haemonchus* spp. and *Mecistocirrus digitatus*. The ITS-1, COX1, and ND4 markers provided sufficient resolution to differentiate *A. sidemi* from other Trichostrongylidae species, whereas the 18S and 28S rRNA genes were too conserved to discriminate species- or subfamily-level relationships. Our findings demonstrate the utility of nuclear and mitochondrial markers for the identification and phylogenetic classification of trichostrongyloid nematodes. These results provide important insights into the invasion dynamics of *A. sidemi*, which may support parasite surveillance and conservation efforts of endangered host species.

## Highlights

• Lack of detectable genetic variability in *Ashworthius sidemi* from two neighbouring European bison populations in Poland.
• Nuclear and mitochondrial markers suggest a single introduction event of *A. sidemi* in Poland.
• Nuclear ITS-1 and mitochondrial COX1 and ND4 enable species-level identification of *A. sidemi* within the Trichostrongylidae.

## Graphical abstract

**Figure:**
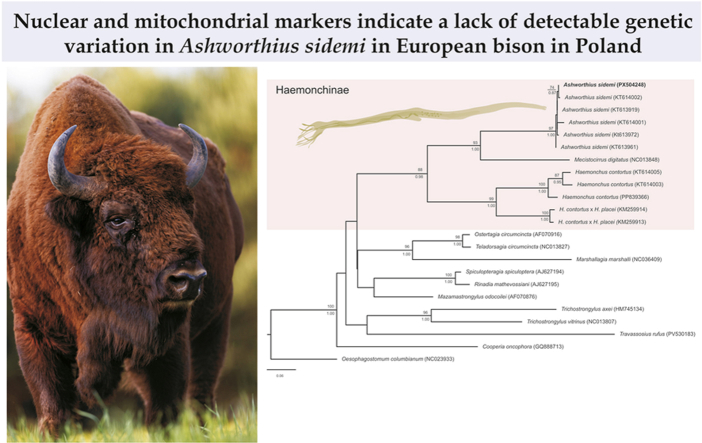

## 1 Introduction

Non-native mammal species introduced through anthropogenic activities may carry and spread new pathogens to new geographical areas. There are numerous examples of parasitic nematodes introduced to Europe along with invasive mammal species, such as *Strongylus robustus*, which naturally infects American grey squirrels (*Sciurus carolinensis*; Romeo et al., 2021) or *Baylisascaris procyonis*, a gastrointestinal roundworm of the raccoon (*Procyon lator*; Osten-Sacken et al., 2018). The blood-sucking nematode *Ashwothius sidemi* Schulz (1933; subfamily Haemonchinae) originally infected Asian deer species, especially sika deer (*Cervus nippon*). Human-mediated translocations of Dybowski's sika deer, a subspecies of *C*. *nippon* (*C*. *n*. *hortulorum*), into central Russia and Ukraine facilitated the westward spread of the parasite in the 20th century. Currently, *A*. *sidemi* is found in all native and non-native cervid species in Europe (Rehbein et al., 2025). The migratory behaviour of the red deer likely facilitated the spread to bovine hosts, including the endangered European bison (*Bison bonasus*) and Tatra chamois (*Rupicapra rupicapra tatrica*; Dróżdż et al., 2003; Nosal et al., 2021).

The European bison (*Bison bonasus* L., 1758), the largest terrestrial mammal in Europe, was extirpated in the wild at the beginning of the 20th century and later restored from captive survivors (Tokarska et al., 2011). The first European bison were released into the wild in the Białowieża Primeval Forest (BPF), and were progressively translocated and introduced to other locations in Eastern Europe (Kowalczyk and Plumb, 2020). Now, there are over 8800 free-ranging European bison worldwide, of which nearly 900 individuals inhabit the BPF in Poland, constituting the largest bison population in the wild, and over 300 live in the Knyszyńska Forest (KF) (Raczyński, 2024). The first occurrence of *A*. *sidemi* in the European bison living in the wild in Poland was found in 1997, in the Bieszczady Mountains (Dróżdż et al., 1998). In 2000, the parasite was already present in bison in BPF (Dróżdż et al., 2003), with a prevalence of 100% estimated in 2004 (Kołodziej-Sobocińska et al., 2016a). The parasite spread rapidly to other bison populations, posing a threat to individual animals, as infection causes abomasitis (Osińska et al., 2010) and a decrease in the number of red blood cells, making them susceptible to other diseases (Kołodziej-Sobocińska et al., 2016b).

Nematodes infecting wild-ranging mobile hosts, such as ungulates, usually have very limited population genetic structure. Using more than one host species usually results in a more genetically unstructured parasite population than infecting a single host (Blouin et al., 1995; Archie and Ezenwa, 2011; Cole and Viney, 2018). Low genetic diversity was often seen in introduced nematode species due to a founder effect, as was observed in *B*. *procyonis* infecting the raccoon in Germany (Osten-Sacken et al., 2018) or in *Passalurus ambiguus* in invasive rabbits (*Oryctolagus cuniculus*) in China (Sheng et al., 2015).

In the last decade, advances in molecular biology have provided a variety of diagnostic markers useful in differentiating trichostrongyle genera infecting wild and domestic ungulates (Eljaki et al., 2016; Avramenko et al., 2018; Davey et al., 2023). Genetic analysis confirmed the presence of *A. sidemi* in samples of European bison faeces (Świsłocka-Cutter et al., 2024) and in other wild ruminants, such as red deer (*Cervus elaphus*), roe deer (*Capreolus capreolus*), fallow deer (*Dama dama*), and mouflon (*Ovis musimon*) (Škorpíková et al., 2025), but still little is known about the genetic variability of this parasite species. A large database of DNA marker sequences with different modes of inheritance, now available in GenBank, is useful in understanding the genetic diversity and evolutionary patterns of invasive nematode species like *A*. *sidemi* and the phylogenetic relationship within the family Trichostrongylidae. In this study, we (1) evaluated the level of genetic variability in *A*. *sidemi* from two neighbouring European bison populations in the BPF and KF using different mtDNA and nDNA markers; (2) determined the taxonomic position of the parasite species within the family Trichostrongylidae based on the available nucleotide sequences in GenBank; and (3) investigated the usefulness of various molecular markers for the taxonomic classification of species from the Trichostrongylidae family.

## 2 Material and methods

### 2.1 Study area

The study was conducted in two European bison habitats located in northeastern Poland: the Białowieża Primeval Forest and the Knyszyńska Forest. The Knyszyńska Forest (53°02′–53°25′ N; 22°55′–23°47′ E) covers approximately 1050 km^2^ of coniferous and mixed forests (Sokołowski, 2006). During the study period, the bison population consisted of 158 individuals in both 2016 and 2017 (Raczyński, 2017, 2018). The area utilized by bison in KF encompasses 1010 km^2^ of forest and adjacent agricultural lands (Kuemmerle et al., 2018). The Polish part of the Białowieża Primeval Forest (52°29′–52°57′ N; 23°31′–24°21′ E) spans 650 km^2^ and is considered one of the best-preserved lowland deciduous forests in Europe (Jędrzejewska and Jędrzejewski, 1998). During the study period, the bison population increased from 596 individuals in 2016 to 654 in 2017 (Raczyński, 2017, 2018). The distribution range of this population covers 690 km^2^ (Kuemmerle et al., 2018). Abomasa samples were collected from European bison culled in November and December of 2016 and 2017 during the winter season.

### 2.2 Genetic analyses and DNA sequencing

An *A*. *sidemi* individual was randomly collected from the abomasum of each of the 16 European bison from the free-ranging populations in the KF (N = 11) and the BPF (N = 5) in 2016 and 2017. The collected material was preserved in sterile 0.2 mL tubes with 70% ethanol at −20°C. In 2021, the worms were transferred to new sterile 0.2 mL tubes and stored at −20°C. These conditions allow for the preservation of DNA suitable for further molecular investigation. Genomic DNA from *A. sidemi* was isolated in 2024 using the DNeasy Blood & Tissue Kit (Qiagen, Hilden, Germany) following the manufacturer's protocol. The extracted DNA was stored at −20°C.

Fragments of three nuclear markers: 18S ribosomal RNA (18S rRNA), 28S ribosomal RNA (28S rRNA), and internal transcribed spacer 1 (ITS-1), and two mitochondrial genes: cytochrome *c* oxidase subunit I (COX1) and NADH dehydrogenase subunit 4 (ND4), were amplified using polymerase chain reaction (PCR) protocols and directly sequenced after purification. PCR amplification of particular markers was carried out with a Labcycler Gradient (SensoQuest, Göttingen, Germany) in 5 μL volumes, and each of the five reaction mixtures consisted of ∼25 ng extracted genomic DNA of *A*. *sidemi* as a template, 1.7 μL of Qiagen Multiplex PCR Master Mix (1×), 0.3 μL mix of appropriate primers (Table 1), and 1 μL of Qiagen nuclease-free water. Each of the five primer mixes contained 0.052 μl of forward and reverse primer (100 pmol/μl). The amplification reactions for the particular markers were performed according to the PCR profile, which included an initial denaturation at 95°C for 15 min, followed by 40 cycles of the PCR reaction consisting of three steps: denaturation at 94°C for 30 s, primer annealing at 57°C (for 18S rRNA, 28S rRNA, ITS-1, ND4) or 48°C (for COX1) for 90 s, and elongation at 72°C for 60 s. At the end, the final elongation phase was performed at 60°C for 30 min. After the PCR, the products were purified with EPPiC Fast (A&A Biotechnology) with an enzymatic reaction according to the manufacturer's protocol. Using forward primers, cycle sequencing PCR was then performed with the BigDye Terminator v. 3.1 Cycle Sequencing Kit (Applied Biosystems, Foster City, CA, USA). Unincorporated dideoxynucleotides were removed from the sequencing reaction using the ExTerminator Kit (A&A Biotechnology). DNA sequencing was performed on an ABI PRISM 3100 Genetic Analyzer (Applied Biosystems, Foster City, CA, USA) automated capillary sequencer.

**Table 1:** Molecular markers and primer sequences used to amplify five chosen DNA fragments in *Ashworthius sidemi*. Tm – primer melting temperature.

Marker	PCR primer sequence 5′–3′	Annealing °C	Primer Tm (°C)	Product length (bp)	References
*Nuclear DNA*					
18S rRNA	F-CAAGGACGAAAGTTAGAGGTTC	57°C	53.2	415	Kobylinski et al. (2012)
	R-GGAAACCTTGTTACGACTTTTA		50.8		
28S rRNA	F-ACAAGTACCGTGAGGGAAAGTTG	57°C	56.9	346	De Ley et al. (2005)
	R-TCGGAAGGAACCAGCTACTA		54.3		
ITS-1	F-GAGAGGACTGCGGACTGCTGTATCG	57°C	62.3	289	Yamada et al. (2012)
	R-AACAACCCTGAACCAGACGT		56.2		
*Mitochondrial DNA*					
COX1	F-TITCIACIAAYCAYAARGAYATTGG	48°C	54.3	418	Geller et al. (2013)
	R-TAIACYTCIGGRTGICCRAARAAYCA		58.6		
ND4	F-GCTTTTATCATTAAGGTTGATAT	57°C	46.8	408	Lehrter et al. (2016)
	R-CWTTRGCWGCTTATTCTTC		43.6		

### 2.3 Statistical and phylogenetic analyses

Sequences of the nuclear 18S rRNA, 28S rRNA and ITS-1 fragments and the mitochondrial COX1 and ND4 gene fragments were edited and aligned manually using BioEdit v. 7.0.5.3 (Hall, 1999). Each indel was manually checked against the original chromatograms to correct potential errors in automated sequencing. All haplotypes generated in this study were deposited in GenBank under the following accession numbers: 18S rRNA (PZ369170), 28S rRNA (PZ369171), ITS-1 (PZ369172), COX1 (PX435475), and ND4 (PX504248). Novel haplotypes of *A. sidemi*, not previously reported in the literature, were identified in the COX1 and ND4 datasets.

To test the evolutionary relationships among the *A. sidemi* haplotypes derived in this study and sequences representing different species from the Trichostrongylidae family downloaded from GenBank, a phylogenetic trees for18S rRNA, 28S rRNA, ITS-1, COX1 and ND4 sequences were constructed. Sequences of different species within the family were retrieved from the NCBI GenBank in July 2025. The search strategy was based on the taxonomic framework available in the NCBI Taxonomy, using the taxonomic identifier (TaxID) for Trichostrongylidae to identify all taxa within this family. Marker-species searchers were then performed for the following loci: 18S rRNA, 28S rRNA, ITS-1, COX1, and ND4. Sequences were excluded if they were incomplete, of low quality or contained ambiguous nucleotides, lacked species-level identification, or were redundant. When multiple sequences were available, preference was given to the longest and best-annotated records.

The multiple sequence alignment program MAFFT version 7 (https://mafft.cbrc.jp/alignment/server/index.html) was used to align the ITS-1 sequences of different species within the Trichostrongylidae family. Phylogenetic relationships for all analysed markers were inferred using both Bayesian Inference (BI) and Maximum Likelihood (ML) approaches. Sequence alignments were prepared in nexux format and used as input for all analyses. Bayesian phylogenetic analyses were conducted using MrBayes v.3.2.7 (Ronquist et al., 2012). The nucleotide substitution models GTR + G (for ITS-1 and ND4), and GTR + G + I (for 18Sr rRNA, 28S rRNA, and COX1) were identified as the best-fitting models according to the Akaike information criterion (AIC), as implemented in jModelTest v.0.1.1 (Posada, 2008). Two independent Markov Chain Monte Carlo (MCMC) runs, each consisting of four chains (one cold and three heated), were performed for 5,000,000 generations, with trees sampled every 1000 generations and a print frequency of 1000. Convergence between runs was assessed using the average standard deviation of split frequencies (ASDSF), which reached values between 0.003 (for 28S rRNA and ND4) and 0.005 (for 18S rRNA). The first 25% of sampled trees were discarded as burn-in, and the remaining trees were used to construct a 50% majority-rule consensus tree. Bayesian posterior probabilities were calculated for each node.

Maximum Likelihood (ML) analyses were performed using IQ-TREE v.3 (Wong et al., 2026). The best-fitting nucleotide substitutions model was selected using ModelFinder based on the Bayesian Information Criterion (BIC). Branch support was assessed using 1000 ultrafast bootstrap (UFBoot) replicates and 1000 SH-like approximate likelihood ratio test (SH-aLRT) replicates. The resulting and Bayesian ML trees were visualized using FigTree v.1.4.4 (http://tree.bio.ed.ac.uk/software/figtree). Outgroup species were selected from the available sequences of the *Rhabditida* order for specific marker analyses. The phylogenetic trees were rooted using outgroup species. *Oesophagostomum columbianum* was used as the outgroup for ITS-1, ND4, 18S rRNA, and COX1 analyses, whereas *O*. *muntiacum* was used for the 28S rRNA dataset. Both taxa represent closely related lineages within Stongylida, outside the family Trichostrongylidae. Their selection was based on the availability of homologous sequences for all analysed genetic markers and comparable alignment lengths for the targeted regions. Branch lengths in the phylogenetic trees are presented as substitutions per site. Pairwise distances between the described haplotypes of *A*. *sidemi* and the Trichostrongylidae species sequences downloaded from the GenBank were calculated using the *p*-distance method in MEGA v.11.0.13 (Tamura et al., 2021).

### 2.4 Ethics statement

No European bison were killed for the purpose of this study. Abomasa samples were collected from animals culled by the authorities managing European bison in Białowieża National Park in BPF, and the Knyszyńska Forest District in KF with permission from the General Directorate for Environmental Protection. The animals were culled for various reasons, including involvement in road accidents or conflicts between individuals, injuries, sickness, and population control. The culling of and sampling from European bison, a protected species, is regulated by the Polish Nature Conservation Act adopted on 16 April 2004 (Journal of Laws no 92/2004, item 880).

## 3 Results

### 3.1 Phylogenetic analysis of nuclear markers

Amplification of the 18S rRNA, 28S rRNA, and ITS-1 regions of the DNA of the 16 individuals yielded fragments of 415, 346, and 289 bp, respectively. A single haplotype was identified for each of the three analysed markers among *A*. *sidemi* individuals.

**Figure S1:**
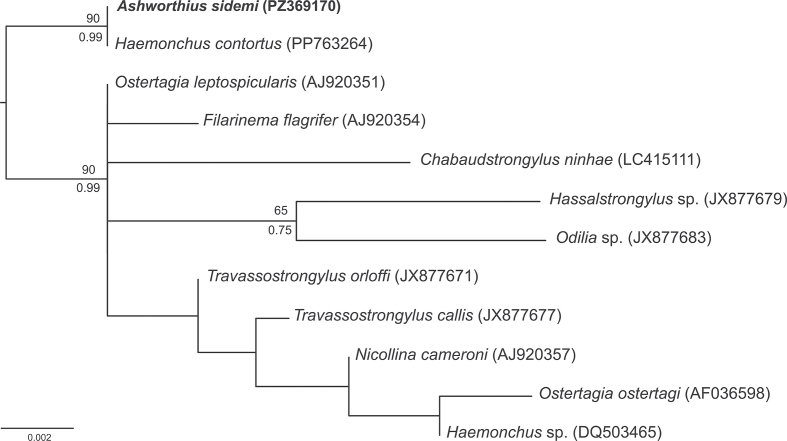
Maximum likelihood phylogenetic tree of Trichostrongylidae haplotypes inferred from the partial nuclear 18S ribosomal RNA (18S rRNA) gene region using IQ-TREE v.3. Branch support values are shown at each node: values above the branches indicate ML support estimated by ultrafast bootstrap (UFBoot) and SH-aLRT (where applicable), while values below the branches represent Bayesian posterior probabilities obtained from MrBayes v.3.2.7. The haplotype of *A*. *sidemi* obtained in this study (GenBank accession no. PZ369170) is marked in bold. Numbers after species names show the accession numbers of the sequences downloaded from GenBank. ML analysis was performed under the best-fitting nucleotide substitution model selected using ModelFinder. The topology is congruent with the Bayesian phylogeny inferred independently in MrBayes. The Bayesian tree is based on the GTR + G + I model of molecular evolution. Scale The scale bar indicates the number of substitutions per site.

The 18S rRNA haplotype (GenBank accession no. PZ369170) found in our study was identical to that previously described in *A. sidemi* from European bison in Poland (GenBank accession no. OP320470; unpublished data) and in *Haemonchus contortus* from alpaca (*Vicugna pacos*) in Poland (GenBank accession no. PP763264; unpublished data). The phylogenetic tree constructed for the family revealed only minor differences among species within the analysed gene region (Supplementary Material: Fig. S1). Pairwise distances between *A. sidemi* and other members of the Trichostrongylidae family ranged from 0.005 (compared to *Ostertagia leptospicularis*; GenBank accession no. AJ920351, Chilton et al., 2006) to 0.015 (compared to *Hassalstrongylus* sp.; GenBank accession no. JX877679, Scheibel et al., 2014). These were the lowest and highest pairwise distances observed among all analysed Trichostrongylidae species.

**Figure S2:**
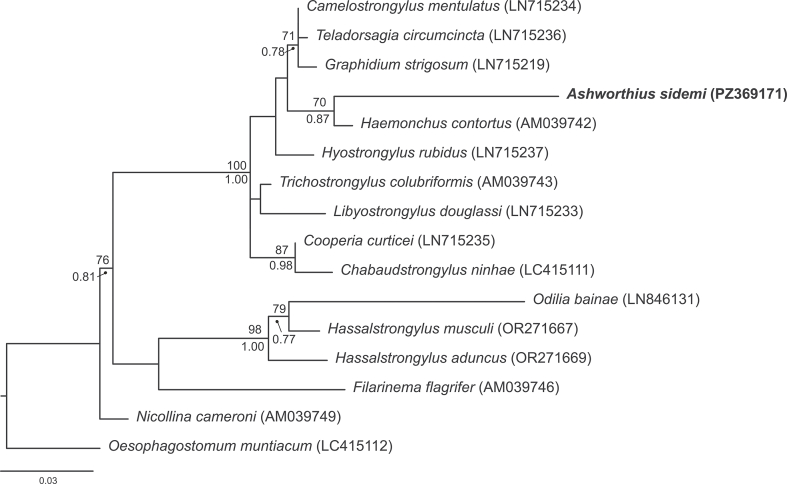
Maximum likelihood phylogenetic tree of Trichostrongylidae haplotypes inferred from the partial nuclear 28S ribosomal RNA (28S rRNA) gene region using IQ-TREE v.3. Branch support values are shown at each node: values above the branches indicate ML support estimated by ultrafast bootstrap (UFBoot) and SH-aLRT (where applicable), while values below the branches represent Bayesian posterior probabilities obtained from MrBayes v.3.2.7. The haplotype of *A*. *sidemi* obtained in this study (GenBank accession no. PZ369171) is marked in bold. Numbers after species names show the accession numbers of the sequences downloaded from GenBank. ML analysis was performed under the best-fitting nucleotide substitution model selected using ModelFinder. The topology is congruent with the Bayesian phylogeny inferred independently in MrBayes. The Bayesian tree is based on the GTR + G + I model of molecular evolution. The scale bar indicates the number of substitutions per site.

The 28S rRNA haplotype (GenBank accession no. PZ369171) identified in the analysed *A. sidemi* individuals matched a haplotype previously found from a roe deer host (GenBank accession no. AF210027; de Bellocq et al., 2001). In the phylogenetic tree, it clustered with *H*. *contortus*, indicating a close evolutionary relationship between the two species (Supplementary Material: Fig. S2). The pairwise genetic distance between them was still low (0.067), and within the family Trichostrongylidae, the two species formed a clade along with *Camelostrongylus mentulatus*, *Chabaudstrongylus ninhae*, *Cooperia curticei*, *Graphidium strigosum*, *Hyostrongylus rubidus*, *Libyostrongylus douglassi*, *Teladorsagia circumcincta*, and *Trichostrongylus colubriformis*.

**Fig. 1:**
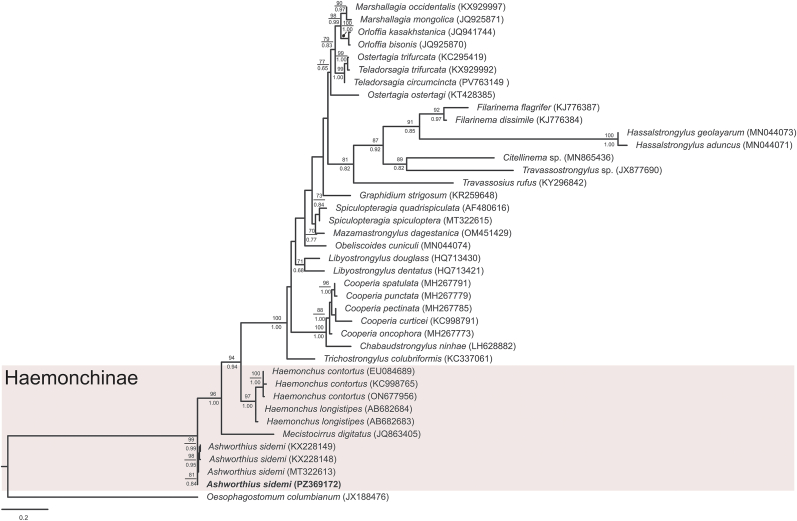
Maximum Likelihood (ML) phylogenetic tree of Trichostrongylidae haplotypes inferred from the partial nuclear internal transcribed spacer 1 (ITS-1) region using IQ-TREE v.3. Branch support values are shown at each node: values above the branches indicate ML support estimated by ultrafast bootstrap (UFBoot) and SH-aLRT (where applicable), while values below the branches represent Bayesian posterior probabilities obtained from MrBayes v.3.2.7. The haplotype of *A*. *sidemi* obtained in this study (GenBank accession no. PZ369172) is marked in bold. Numbers after species names show the accession numbers of the sequences downloaded from GenBank. The ML analysis was performed under the best-fitting nucleotide substitution model selected using ModelFinder. The topology is congruent with the Bayesian phylogeny inferred independently in MrBayes. The Bayesian tree is based on the GTR + G model of molecular evolution. The scale bar indicates the number of substitutions per site.

The ITS-1 haplotype (GenBank accession no. PZ369172) was identical to that previously described for *A. sidemi* (GenBank accession no. EF467325; unpublished data) and clustered with other *A. sidemi* haplotypes in the phylogenetic tree (Fig. 1), although the lack of publicly available voucher material for this reference should be noted. Pairwise distances between the haplotype from this study and three other *A. sidemi* haplotypes ranged from 0.003 (GenBank accession no. MT322613, Kuznetsov et al., 2020) to 0.010 (GenBank accession no. KX228149, Skorpikova et al., 2020). Although *A. sidemi* and *Mecistocirrus digitatus* clustered within the same clade, the observed ITS-1 genetic distance (0.180) suggested that they represent clearly distinct evolutionary lineages. In the phylogenetic tree, these two species clustered with *H*. *contortus* and *H. longistipes*. Pairwise distances among haplotypes of *A*. *sidemi*, *M*. *digitatus* and *Haemonchus* spp. ranged from 0.003 to 0.189.

### 3.2 Phylogenetic analysis of mitochondrial genes

Amplification of the mitochondrial COX1 and ND4 genes yielded fragments of 418 bp and 408 bp, respectively. A single haplotype was identified for each of the two genes among the 16 *A. sidemi* individuals. The resulting phylogenetic trees supported the findings obtained from nuclear markers.

**Figure S3:**
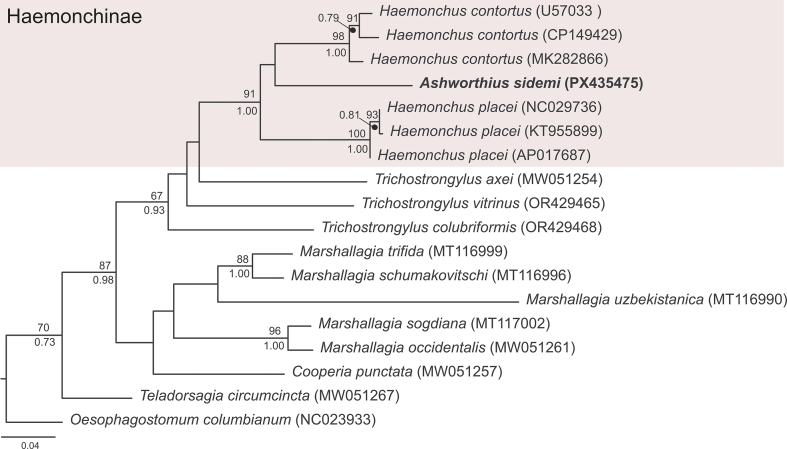
Maximum likelihood phylogenetic tree of Trichostrongylidae haplotypes inferred from the partial mitochondrial cytochrome *c* oxidase subunit I (COX1) gene region using IQ-TREE v.3. Branch support values are shown at each node: values above the branches indicate ML support estimated by ultrafast bootstrap (UFBoot) and SH-aLRT (where applicable), while values below the branches represent Bayesian posterior probabilities obtained from MrBayes v.3.2.7. The haplotype *A*. *sidemi* obtained in this study (GenBank accession no. PX435475) is marked in bold. Numbers after species names show the accession numbers of the sequences downloaded from GenBank. ML analysis was performed under the best-fitting nucleotide substitution model selected using ModelFinder. The topology is congruent with the Bayesian phylogeny inferred independently in MrBayes. The Bayesian tree is based on the GTR + G + I model of molecular evolution. The scale bar indicates the number of substitutions per site.

The analysis of the COX1 gene fragment produced a novel sequence, representing the first COX1 sequence described for *A. sidemi* (GenBank accession no. PX435475). In the phylogenetic tree, this sequence clustered within the clade containing *H*. *placei* and *H. contortus* (Supplementary Material: Fig. S3). Genetic distances between *A. sidemi* and *Haemonchus* species ranged from 0.113 – between *A. sidemi* and *H. contortus* isolated from domestic goat (*Capra hircus*; GenBank accession no. MK282864; unpublished data) – to 0.127, compared to *H. contortus* isolated from sheep (*Ovis aries*; GenBank accession no. EU346694, Jex et al., 2008).

**Fig. 2:**
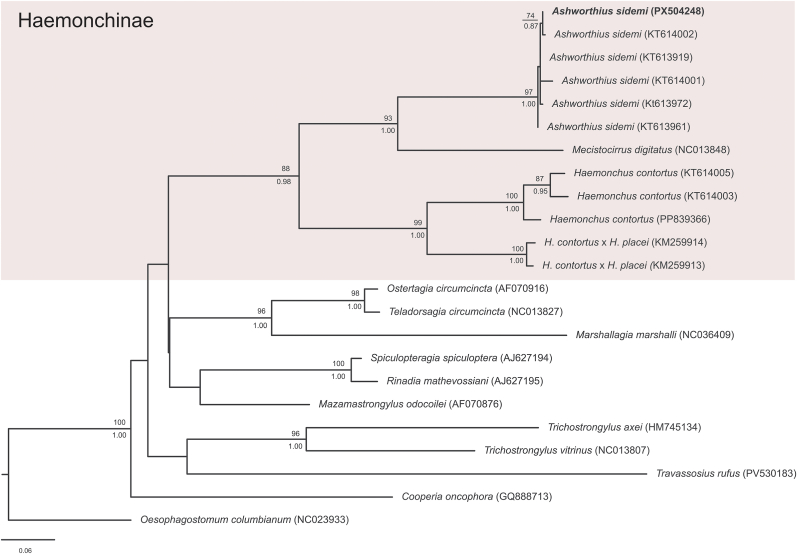
Maximum Likelihood (ML) phylogenetic tree of Trichostrongylidae haplotypes inferred from the partial mitochondrial NADH dehydrogenase subunit 4 (ND4) gene region using IQ-TREE v.3. Branch support values are shown at each node: values above the branches indicate ML support estimated by ultrafast bootstrap (UFBoot) and SH-aLRT (where applicable), while values below the branches represent Bayesian posterior probabilities obtained from MrBayes v.3.2.7. The haplotype of *A*. *sidemi* obtained in this study (GenBank accession no. PX504248) is marked in bold. Numbers after species names show the accession numbers of the sequences downloaded from GenBank. The ML analysis was performed under the best-fitting nucleotide substitution model selected using ModelFinder. The topology is congruent with the Bayesian phylogeny inferred independently in MrBayes. The Bayesian tree is based on the GTR + G model of molecular evolution. The scale bar indicates the number of substitutions per site.

The ND4 gene analysis revealed a previously undescribed haplotype of *A. sidemi* (GenBank accession no. PX504248). The Polish ND4 haplotype differed from the French *A*. *sidemi* haplotypes obtained from nematodes isolated from European roe deer in France (*Capreolus capreolus*; Lehrter et al., 2016) by 1–6 polymorphic sites. The closest haplotype (KT614002) differed by a single transition at position 6 (A/G; pairwise distance = 0.002), whereas the most divergent haplotype (KT614001) showed six substitutions at positions 31 (C/T), 49 (A/G), 61 (T/C), 312 (A/G), 358 (T/A), and 367 (T/A; pairwise distance = 0.015). Haplotypes KT613919, KT613961, and KT613972 differed by two substitutions each, located at positions 312 (A/G) and 358 (T/C), positions 91 (A/G) and 312 (A/G), and positions 312 (A/G) and 358 (T/C), respectively. Similarly to the phylogenetic tree based on the ITS-1 marker, *A. sidemi* formed a clade with *M*. *digitatus* in the mitochondrial ND4 analysis, with a genetic distance of 0.179 between the two species (Fig. 2). These parasite species, in turn, formed a strongly supported clade with *H*. *contortus* and *H. contortus × H. placei* hybrids.

### 3.3 Limitations of sampling

While the phylogenetic analyses indicated no intraspecific variation across markers, the sampling design imposes certain limitations. To assess the robustness of our sampling design, we acknowledge that sequencing a higher number of *A*. *sidemi* individuals per European bison host would have provided greater statistical power to detect within-host genetic variation. However, given the limited sample size per host in the present study, our ability to detect additional haplotypes in the analysed nuclear and mitochondrial markers, as well as low-frequency genetic variants, remains constrained. Consequently, although the observed absence of intraspecific variation across all analysed markers supports the hypothesis of a genetically homogeneous *A*. *sidemi* in the studied area, we cannot exclude the presence of rare or unsampled haplotypes.

## 4 Discussion

Our study revealed no detectable genetic variability among specimens of the ecologically invasive blood-sucking nematode *A*. *sidemi* sequenced for the analysed nuclear and mitochondrial markers across two neighbouring European bison populations in BPF and KF in northeastern Poland. Originally a parasite of Asian cervids, *A*. *sidemi* has progressively spread across Europe since the second half of the 20th century (Rehbein et al., 2025). While infections usually involve only a few hundred nematode individuals in a single host, intensities can reach tens of thousands in non-specific hosts such as the European bison (Demiaszkiewicz et al., 2008, 2009a).

*A*. *sidemi* was first detected in the BPF European bison population in 2000 (Dróżdż et al., 2003), while the first record in the neighbouring KF, located at a minimum distance of approximately 60–70 km away, was reported in 2009 (Demiaszkiewicz et al., 2009b). The KF population was established through one male that migrated from BPF and six individuals translocated from BPF in 1974, which formed the foundation of the current population. The presence of *A*. *sidemi* in KF may have been associated with the translation of two young females from captivity in 2005 (Bozik, 2008; Demiaszkiewicz et al., 2009b; Kołodziej-Sobocińska et al., 2023), although direct parasitological confirmation of infection at the time of translocation is lacking. Therefore, this scenario should be considered an interpretation inferred from subsequent molecular and epidemiological observation rather than a confirmed introduction event. However, natural parasite dispersal through movements of wild cervids between BPF and KF cannot be completely excluded. The apparent delay between the presumed introduction and first detection in 2009 may additionally reflect low initial infection prevalence and limitations in early parasitological surveillance. Given that ungulates are mobile and capable of dispersing over large distances, both natural movements and anthropogenic translocations likely facilitate gene flow among parasite populations. This may contribute to the genetic homogeneity of *A*. *sidemi* observed between these two geographically close European bison populations in Poland.

From a One Health perspective, the continued expansion of *A*. *sidemi* in wild cervid populations may have important implications for sympatric domestic ruminants. Previous studies have confirmed the presence of *A*. *sidemi* DNA in cattle grazing in areas inhabited by infected wildlife, including the buffer zone of the Białowieża Primeval Forest and the Strzałowo Forest District in Poland, supporting the possibility of spillback transmission at the wild-livestock interface (Moskwa et al., 2015). Although the epidemiological significance and pathogenic effects of *A*. *sidemi* infection in cattle remain poorly understood, the close phylogenetic and biological relationship between *A*. *sidemi* and the hematophagous nematode *H*. *contortus* raises concerns regarding its potential veterinary importance. Given the blood-feeding behavior of both species, heavy infections of *A*. *sidemi* could potentially contribute to anemia, reduced productivity, and impaired health status in susceptible hosts (Moskwa et al., 2015; Kornacka et al., 2020). These findings highlight the need for continued surveillance of *A*. *sidemi* in both wildlife and livestock populations, particularly in regions where extensive grazing systems facilitate contact between wild and domestic ruminants.

No detectable intraspecific variability among *A*. *sidemi* specimens from European bison populations in Poland is consistent with general trends observed in invasive nematodes, where host population bottlenecks and anthropogenic translocations can lead to reduced parasite genetic diversity (Cole and Viney, 2018). Alien species typically undergo a genetic bottleneck during introduction, a pattern commonly reported in parasitic nematodes. Similar trends have been observed in *Baylisascaris procyonis* infecting the raccoons in Germany (Osten-Sacken et al., 2018) and *Passalurus ambiguous* parasitising European rabbits in China (Sheng et al., 2015). *A*. *sidemi* was introduced into Europe via sika deer, with early reports from Ukraine, Slovakia, Czechia, and France (Ovcharenko, 1968; Kotrla and Kotrly, 1973, 1977; Ferté et al., 2000). Dróżdż et al. (2003) proposed that the source of this nematode species in Poland was local deer populations in the Bieszczady Mountains, which may have acquired the parasite from neighbouring Ukraine and Slovakia. The limited genetic diversity of *A. sidemi* observed in this study may therefore be a consequence of introduction from a single source population and/or a single host species, combined with ongoing gene flow via cervid reservoirs, which could contribute to homogenisation of the parasite's genetic structure. The presence of several mitochondrial ND4 haplotypes in French populations of *A. sidemi* is consistent with multiple introduction events of the parasite via sika deer originating from three geographically distinct regions: Japan (*C. n. nippon*), Manchuria (*C. n. mantchuricus*), and Tonkin (*C. n. hortulorum*) (Beaufort, 1984).

The family Trichostrongylidae (Nematoda: Strongylida), to which *A*. *sidemi* belongs, is a diverse group of gastrointestinal parasites commonly infecting ruminants (Anderson et al., 1998). It includes six subfamilies: Trichostrongylinae, Libyostrongylinae, Haemonchinae, Cooperiinae, Ostertagiinae, and Graphidiinae (Gibbons and Khalil, 1982). While some species within Trichostrongylidae exhibit high host specificity, others, such as *A*. *sidemi*, can infect a broad range of wild and domestic ungulates (Rehbein et al., 2025; Škorpíková et al., 2025). Notably, many species within the family are characterized by substantial genetic diversity, with multiple studies documenting cryptic species, genetic lineages, and species complexes (Blouin, 2002; Leignel et al., 2002; Grillo et al., 2007; Cerutti et al., 2010). Despite the growing epidemiological importance of *A*. *sidemi*, molecular studies on this species are still scarce. One of the few genetic investigations, based on the mitochondrial ND4 gene, enabled clear differentiation between *A. sidemi* and the closely related *H*. *contortus*, even in morphologically indistinct stages (Lehrter et al., 2016). Nevertheless, many aspects of the species' genetics remain unexplored, particularly large-scale analyses of nuclear and mitochondrial markers from geographically diverse populations. This gap hampers our understanding of the species’ phylogeography, genetic structure, and transmission dynamics.

Even with the expanding availability of molecular tools, phylogenetic relationships within Trichostrongylidae remain only partially resolved. Traditional taxonomy based on morphological traits is frequently contradicted by genetic analyses, which often reveal phylogenetic clustering across subfamilies or genera (Blouin, 2002; Chilton et al., 2006). Molecular studies employing both nuclear and mitochondrial markers have greatly advanced our understanding of the evolutionary relationships within the family. In particular, these tools have enabled the resolution of species complexes in *Haemonchus*, *Trichostrongylus*, and *Ashworthius* (Leignel et al., 2002; Cerutti et al., 2010; Chaudhry et al., 2016). Phylogenetic analyses further indicate that some lineages exhibit patterns consistent with host-associated diversification, whereas others suggest host-switching, likely driven by ecological overlap or anthropogenic activity (Hoberg and Brooks, 2008). These evolutionary relationships have important implications for taxonomy, and understanding them is crucial in studies of parasite ecology, biogeography, and host-parasite coevolution. In the case of *A. sidemi*, the available data did not allow assessment of a potential association between phylogenetic lineage diversity and host species, due to the recovery of a single haplotype. This lack of detectable structure may indicate relatively low genetic variability within the species.

Both morphological and molecular studies have demonstrated the taxonomic complexity within Trichostrongylidae and the limitations of relying solely on morphology for species identification. This is particularly evident in the subfamily Haemonchinae, where accurate identification is feasible only in adult males. Females, immature individuals, eggs, and larvae are often morphologically indistinguishable between species (Lehrter et al., 2016). *Haemonchus* spp. and *A*. *sidemi* are prominent examples of cryptic species complexes, consisting of morphologically similar but genetically distinct taxa (Lehrter et al., 2016). These diagnostic challenges hinder effective monitoring and control of trichostrongyloid infections. Therefore, molecular approaches, especially DNA sequencing, are essential for precise species identification and the detection of cryptic diversity.

The selection of appropriate molecular markers is critical in resolving species identification and uncovering hidden diversity within Trichostrongylidae. In the present study, the most notable result of the 18S rRNA analysis was the identical haplotype shared between *A*. *sidemi* and *H*. *contortus* from alpaca in Poland. This findings highlights the limited resolving power of the 18S rRNA marker for distinguishing closely related taxa within Haemonchinae. Accordingly, the nuclear 18S rRNA gene fragment showed insufficient variation to discriminate between *Haemonchus* spp. and *A*. *sidemi* in our phylogenetic analyses, indicating a high level of sequence conservation across these genera. Consequently, although the 18S rRNA gene is valuable for broader-scale phylogenetic inference, it appears insufficient for species-level discrimination within this group, consistent with previous observations in strongylid nematodes. This limitation reflects the relatively slow evolutionary rate of the 18S rRNA gene, which restricts its utility for resolving closely related species within the family Trichostrongylidae (Blouin et al., 1998; Gasser, 2006). Extending beyond species-level resolution, the phylogenetic tree constructed in this study did not support the monophyly of the six currently recognized subfamilies of Trichostrongylidae, suggesting that 18S rRNA does not provide sufficient phylogenetic signal for resolving deeper taxonomic relationships within the group. Overall, the limited resolving capacity of 18S rRNA highlights the importance of multilocus approaches in the phylogenetic analysis of trichostrongyloid nematodes, particularly when studying species complexes or investigating evolutionary relationships within morphologically conserved taxa.

Our molecular analyses revealed a 28S rRNA haplotype identical to a sequence of *A. sidemi* previously found in the European roe deer (de Bellocq et al., 2001). Its close relationship with *H*. *contortus* (pairwise distance = 0.067) is consistent with earlier findings placing *Ashworthius* and *Haemonchus* within the subfamily Haemonchinae, highlighting their evolutionary proximity. However, consistent with previous studies, the 28S marker also showed limited resolution at higher taxonomic levels, failing to clearly distinguish subfamilies within Trichostrongylidae (Blouin, 2002; Chilton et al., 2006). These results support the use of multilocus approaches and more variable molecular markers, such as internal transcribed spacers and mitochondrial genes, which provide improved resolution at both species and higher taxonomic levels (Chilton et al., 2006; Hu and Gasser, 2006; Cerutti et al., 2010).

In contrast, the ITS-1 region demonstrated strong discriminatory power. The *A. sidemi* haplotype obtained in this study clustered tightly with other *A. sidemi* sequences, exhibiting low intraspecific divergence (0.003–0.010). The ITS-1 primers described by Yamada et al. (2012), originally developed for Heligmonellidae, were applied due to the conserved nature of ribosomal DNA internal transcribed spacer regions within the superfamily Trichostrongyloidea. ITS regions are widely used molecular markers in nematodes owing to conserved primer-binding sites and sufficient variability for species-level discrimination (Chilton, 2004). The successful amplification of *A*. *sidemi* is consistent with their close phylogenetic relationship to Heligmonellidae and the conserved structure of the rDNA locus across Strongylida (Gasser and Newton, 2000). These results further confirm the utility of the ITS region as a reliable marker for species identification and support the low genetic diversity observed in *A. sidemi*, which may reflect a founder effect among several possible explanations, including a limited host range during its colonization (Kuznetsov et al., 2020; Skorpikova et al., 2020). Furthermore, the placement of *A. sidemi* alongside *M*. *digitatus* and *Haemonchus* species corroborates morphological classifications and supports the hypothesis of a shared evolutionary history among these genera within the Haemonchinae subfamily (Leignel et al., 2002; Chaudhry et al., 2016).

Mitochondrial markers, COX1 and ND4, provided even better phylogenetic resolution within the Trichostrongylidae family. For the first time, a COX1 sequence was obtained for *A. sidemi*, expanding the molecular resources available for this species. Phylogenetic analysis revealed a close relationship with *H*. *contortus* and *H. placei*, with interspecific genetic distances ranging from 0.113 to 0.127. This is consistent with broader nematode studies, where COX1 variability reliably distinguishes species (mean interspecific distances of approximately 10–25%), while showing low intraspecific variability (0.5–3.5%) (Hu and Gasser, 2006; Jex et al., 2008). Similarly, the newly identified ND4 haplotype of *A. sidemi* clustered within a clade including *M*. *digitatus* and *Haemonchus* species. The genetic distance between *A. sidemi* and *M. digitatus* (0.179) further confirms their distinct yet related evolutionary status. Importantly, the relatively low divergence observed between the studied haplotype and those found in individuals infecting *Capreolus capreolus* in France (Lehrter et al., 2016) suggests limited genetic differentiation among the currently available European *A. sidemi* sequences. However, broader geographic sampling is needed to assess the population structure of *A. sidemi* in Europe more comprehensively.

In summary, the consistent topologies obtained from nuclear (ITS-1, 28S rRNA) and mitochondrial (COX1, ND4) markers reinforce the phylogenetic position of *A. sidemi* within the Haemonchinae subfamily and its close affinity to *Haemonchus* spp. and *M*. *digitatus*. Conserved nuclear genes (e.g., 18S rRNA, 28S rRNA) proved useful for broader phylogenetic inference, whereas ITS and mitochondrial markers offered higher discriminatory power for species-level identification. No detectable intraspecific genetic variability was observed among the sequenced specimens from both European bison populations, which may be consistent with a founder effect among several possible demographic scenarios, and highlights the need for continued monitoring of this invasive parasite in endangered host species.

## Funding

This project was partially financed under a subsidy for maintaining the research potential of the Faculty of Biology, University of Bialystok. The material was collected as part of the project no. 2012/07/B/NZ8/00066 financed by the National Science Centre.

## CRediT authorship contribution statement

**Magdalena Świsłocka-Cutter:** Conceptualization, Formal analysis, Funding acquisition, Investigation, Methodology, Project administration, Supervision, Visualization, Writing – original draft. **Anetta Borkowska:** Writing – original draft, Writing – review & editing. **Rafał Kowalczyk:** Supervision, Writing – review & editing. **Marta Kołodziej-Sobocińska:** Investigation, Supervision, Writing – original draft, Writing – review & editing.

## Declaration of competing interest

The authors declare that they have no conflict of interest.
